# Recombinant lactic acid bacteria as promising vectors for mucosal vaccination

**DOI:** 10.1002/EXP.20210026

**Published:** 2021-10-30

**Authors:** Nan Qiao, Guangsheng Du, Xiaofang Zhong, Xun Sun

**Affiliations:** ^1^ Key Laboratory of Drug‐Targeting and Drug Delivery System of the Education Ministry and Sichuan Province Sichuan Engineering Laboratory for Plant‐Sourced Drug and Sichuan Research Center for Drug Precision Industrial Technology West China School of Pharmacy, Sichuan University Chengdu China

**Keywords:** adjuvant, lactic acid bacteria, mucosal immunity, mucosal vaccines

## Abstract

Lactic acid bacteria (LAB), a diverse family of gram‐positive bacteria, has been proven effective in delivering varieties of therapeutic and prophylactic molecules such as antigens and cytokines. Featuring the properties of acid‐resistant, high uptake into Peyer's patches, and superior capacity for inducing secretory IgA antibodies, LAB have good potential to be used as vaccine vectors for mucosal vaccination. Mucosal immunization enables both mucosal and systemic immune responses, which are critical for resisting pathogens that invade the host through the mucosal surfaces. With the development of genetic engineering, LAB strains, primarily *Lactococcus* and *Lactobacillus* have been exploited to express a range of heterologous antigens. Numerous studies have demonstrated that LAB mucosal vaccines can stimulate all arms of the immune system to provide adequate protection against pathogen infections. Additionally, several LAB‐based human *papillomavirus* vaccines have entered the clinical trial studies, which suggest the great promise of LAB vaccines for new interventions in mucosal transport diseases. Herein, we will discuss the factors that influence the immunogenicity of LAB vaccines, including LAB strains, the location of antigens, and administration routes, and focus on the current strategies that have been reported for optimizing LAB vaccines.

## Graphical abstract

Schematic illustration of the locations of expressed antigen by recombinant lactic acid bacteria (LAB) and vaccine strategies applicated in LAB vaccines for enhancing immune responses.

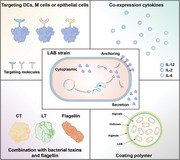

## 1 INTRODUCTION

Lactic acid bacteria (LAB) are gram‐positive, non‐pathogenic, non‐spore‐forming bacteria, and are generally regarded as safe (GRAS) according to American Food and Drug Administration (FDA).^[^ ^1^ ^]^ LAB have been safely used in various fermented foods and beverage for centuries, such as yogurts and cheese. LAB comprise the genera of *Lactococcus*, *Lactobacillus*, *Streptococcus*, *Bifidobacterium*, and several others and among which *Lactobacillus* and *Bifidobacterium*, are considered as probiotics that have multiple benefits to gastrointestinal health.^[^ ^2^ ^]^ The probiotic LAB strains play key roles in improving the digestion and absorption of nutrients, maintaining energy and intestinal microflora homeostasis, and most importantly modulating the immune system.^[^ ^3^ ^]^

**FIGURE 1:**
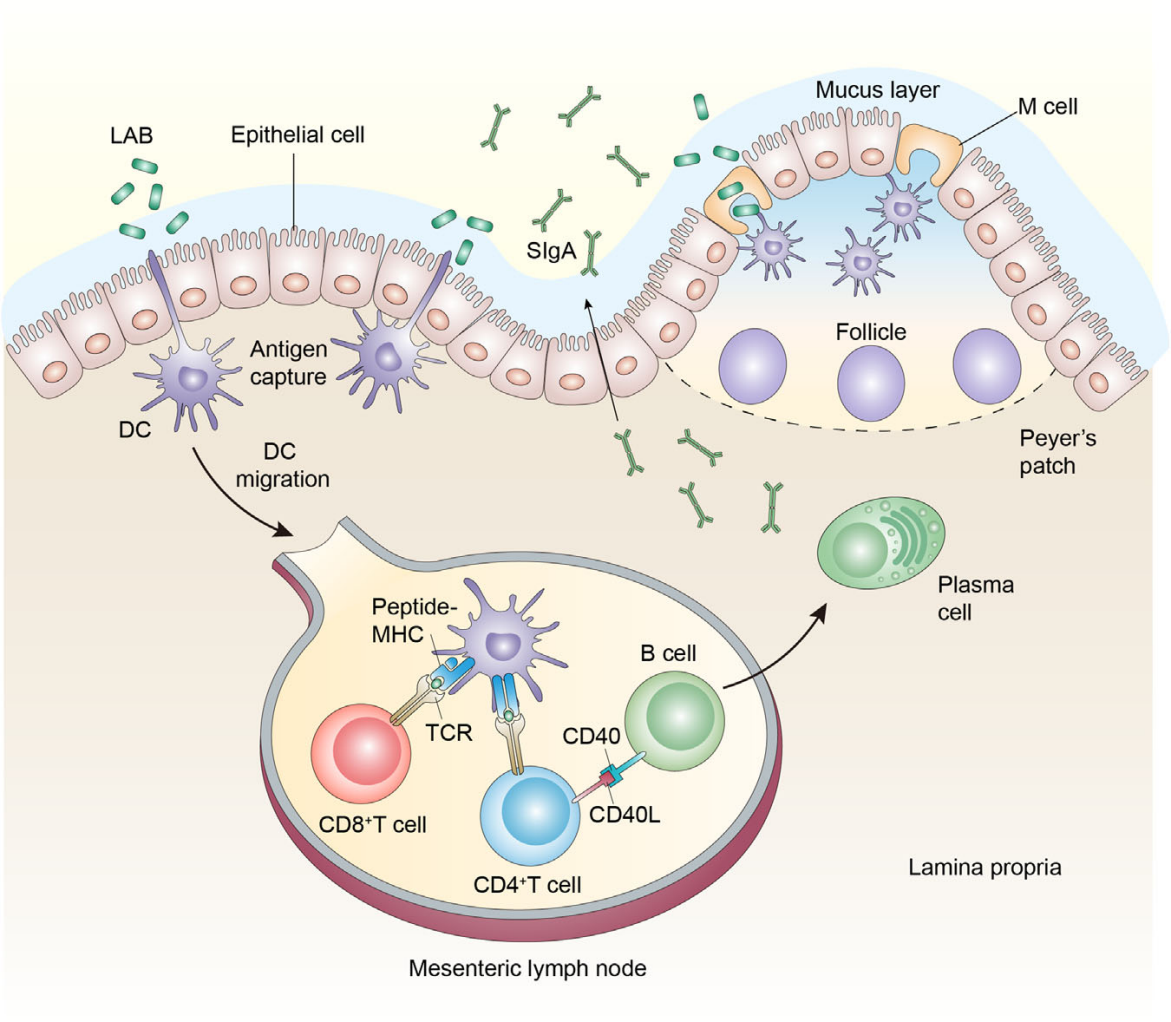
Schematic illustration of mucosal immunization induced by recombinant LAB. Dendritic cells (DCs) phagocytose bacteria that touch the apical surface of epithelial cells by extending transepithelial protrusions or by interacting with M cells, which can transport antigens across the epithelial layer to DCs in the dome of Peyer's patch. After capturing antigens, DCs migrate to mesenteric lymph node, where DCs can directly prime T‐cell responses to antigens and B cell is activated by interaction with T‐helper cells. IgA is generated by mature plasma cells in lamina propria, which has critical functions in persisting pathogens infection

For more than two decades, LAB has been extensively exploited as potential mucosal delivery carriers. Vast pathogens are continuously interacting with mucosal surfaces of host, including gastrointestinal, respiratory, and genitourinary tracts, which lines the first defense against infections. In this respect, it is necessary to design vaccines that could induce a potent mucosal immune response. There are a large number of mucosa‐associated lymphoid tissues (MALT), the most representative of which are gut‐associated lymphoid tissue, nasopharynx‐associated lymphoid tissue, and bronchus‐associated lymphoid tissue.^[^ ^4^ ^]^ Mucosal delivery of vaccines can provoke antigen‐specific secretory immunoglobulin (Ig) A responses at mucosal sites^[^ ^5^ ^]^ and effective systemic immune responses (Figure 1). Besides, mucosal immunization does not require strict control of an extremely low endotoxin content as same as injections^[^ ^6^ ^]^ and professional medical staff to inoculate, which is convenient for vaccination as well as exhibits good compliance, and avoids the risk of blood‐borne diseases related to syringe contamination.^[^ ^7^ ^]^ Therefore, mucosal vaccination is preferred by vaccinees as compared to routine vaccination by injection.

However, despite these advantages of mucosal vaccines, the delivery of soluble antigens through mucosal membranes faces problems of easy degradation and low immunogenicity.^[^ ^8^ ^]^ The development of suitable mucosal delivery vehicles that can target MALT is needed to enhance immunogenicity of the antigen. Currently, the major carriers for mucosal vaccines include liposomes, polymeric nanoparticles or microspheres, and live bacteria or viruses as vectors.^[^ ^9^, ^10^ ^]^ Compared with particle‐mediated delivery systems, live bacteria are more immunogenic due to innate immune responses to pathogens and capable to manipulate the genomes using genetic engineering techniques to express a variety of heterologous antigens.^[^ ^11^ ^]^ However, most bacteria have lipopolysaccharides and other pathogen‐associated molecular patterns, which may cause undesirable side effects especially in young children and immunosuppressed people.^[^ ^10^ ^]^ Even if some bacteria are attenuated by deleting critical pathogenic genes such as *Salmonella typhi Ty21a / galE* ^*−*^, there is still the possibility of reverting to pathogenic bacteria. Therefore, the non‐pathogenic LAB is gradually attracting attention as the mucosal delivery vectors of antigens and therapeutic proteins. LAB offers several attractive superiorities as a mucosal delivery vector, such as the ability to resist the low pH of the gastrointestinal tract and high concentrations of bile salt, easiness for being taken up into Peyer's patches, induction of low‐level immune responses against themselves, and adjuvant properties for certain strains.^[^ ^12^ ^]^

Several critical factors can affect the delivery efficiency of the LAB mucosal vaccine. This review will illustrate the influence of different LAB strains mainly *Lactococcus lactis* (*L. lactis*) and *Lactobacillus*, as well as different antigen locations such cytoplasmic, secreted, and cell‐wall anchored forms on immune responses that can be induced. Moreover, we discuss the mucosal administration routes of LAB vaccines and compare how mucosal administration routes can affect the efficacy of LAB vaccines. Despite the intrinsic adjuvant properties of LAB, published studies have adopted various strategies to enhance the immunogenicity. Here, we will highlight the potential strategies that have been reported to improve the immune efficacy of mucosal delivered LAB vaccines.

## 2 LAB AS VACCINE DELIVERY VEHICLES

### 2.1 The bacterial strains of LAB

Since LAB are non‐invasiveness and non‐pathogenicity, they have been widely used for mucosal vaccine delivery. Among the reported studies, the most popular LAB genera are *L. lactis* and *Lactobacillus*. Both LAB genera are possible to introduce plasmids by genetic tools to express foreign proteins stably. And some strains can colonize in the intestines to evoke a potent immune response. Although some studies have developed Bifidobacterium as vaccine delivery vehicles, the following review will mainly discuss *L. lactis* and *Lactobacillus*.

*L. lactis*,^[^ ^13^ ^]^ particularly the plasmid‐free strains of IL1403 and MG1363, are considered as model microorganisms in LAB research. The development of various genetic tools and the identification of complete genome sequence make it possible to use *L. lactis* to efficiently express heterologous antigens. So far, both constitutive and inducible promoters have been developed for *L. lactis* gene expression system. Different plasmids and promoters in bacterial vehicles have great influence on the production efficiency of heterologous antigens, further priming discrepant immune response intensities. Constitutive promoters such as P3 and P2 are usually strong promoters,^[^ ^14^ ^]^ which can achieve sustained high expression of certain antigens, leading to these antigens accumulation or degradation in cells that could be deleterious to cells. By contrast, inducible promoters are promoters that require an inducer such as pH, temperature, or chemical molecules for gene expression. The most common and effective inducible expression system is the Nisin Induced Controlled Expression system^[^ ^15^ ^]^ that requires nisin as an inducer. In this case, the efficiency of gene expression by changing the amount of added nisin.

In recent years, *Lactobacillus* used as mucosal vehicle has received extensive attention. Unlike *L. lactis*, *Lactobacillus* has diverse bacterial species, and the commonly used ones include *Lactobacillus casei* (*L. casei*), *Lactobacillus plantarum* (*L. plantarum*), and *Lactobacillus rhamnosus*.^[^ ^16^ ^]^ *Lactobacillus* has attractive properties, including 1) the peptidoglycan layer of certain strains has stronger adjuvanticity, 2) some of the strains can colonize in the intestines for a long time,^[^ ^17^ ^]^ all of which are beneficial to evoke a robust immune response. However, it is difficult to use *Lactobacillus* as a vector for gene expression due to its genetic diversity. The activity of plasmid replication system and the promoter depending on the *Lactobacillus* strains. Nevertheless, there are still many studies that showed that *Lactobacillus* as a vector can successfully express foreign antigens.

The strain of bacterial hosts can affect the and type and intensity of immune responses since different strains of bacterial strains differ in their component of the peptidoglycan layer and their capacity of colonizing in the murine gastrointestinal tract or nasal mucosal sites. For example, a model antigen tetanus toxin fragment C (TTFC), a model antigen, has been produced by several different bacterial carriers to investigate the influence of bacteria strains on immunogenicity. For this purpose, recombinant wide‐type *L. lactis* and *L. plantarum* expressing TTFC intracellularly^[^ ^18^ ^]^ were intragastric or intravaginal administered to mice. The level of anti‐TTFC serum IgG titers and neutralizing tetanus antibodies in pooled sera elicited by wild‐type (WT) *L. plantarum* were higher than that elicited by WT *L. lactis* following either intragastric or intravaginal immunization. Additionally, the influence of bacterial strains on immune responses was analyzed in LAB vaccines against HPV‐16.^[^ ^19^ ^]^ The western blot showed that the amount of E7 antigen displayed on the surface of *L. lactis* and *L. plantarum* was equivalent. However, the secretion of serum IgG, IgA, and IFN‐γ could only be observed in mice intranasally immunized by the *L. plantarum* strain. Consequently, compared with *L. lactis*, *L. plantarum* that expressed E7 antigen is more efficient in inhibiting TC‐1 tumors after mice challenged subcutaneously inoculated with TC‐1 tumor cells. In sum, both studies mentioned above indicated that *L. plantarum* has more intrinsic adjuvant potential than the strain of *L. lactis*.

### 2.2 The impact of antigen cellular locations on immunity

Cellular locations of foreign antigens after expression including cytoplasmic, secreted, and cell‐wall anchored forms which differ in the intensity and direction of immune responses. For LAB vaccine carriers, the signal peptide of usp45 generally applied in *L. lactis* and several cell‐wall anchored proteins have been developed to realize the heterologous antigens in a secreted form or a surface‐displayed form. Several studies have compared the impacts of the different locations of antigens on immunity.

For subcutaneous immunization, cytoplasm, secreted outside the cell, and cell‐wall tethered form were compared for A2 antigen from *Leishmania donovani* expressed by *L. lactis*.^[^ ^20^ ^]^ The concentrations of anti‐A2 antibody IgG in the cell‐wall anchored *L. lactis* group was determined to be the highest. Similar research was carried out in the same LAB strain expressing TTFC,^[^ ^21^ ^]^ but mice immunization with the cytoplasmic mode of antigen expression elicited the most robust response. Besides, Reveneau et al. have tested the immunogenicity of *L. plantarum* producing TTFC in three cellular locations and concluded no distinctive difference in immune responses among these groups.^[^ ^22^ ^]^ The different results regarding the cellular positions may be associated with the quantities of antigen expression and the degradation of labile protein in carriers.

**FIGURE 2:**
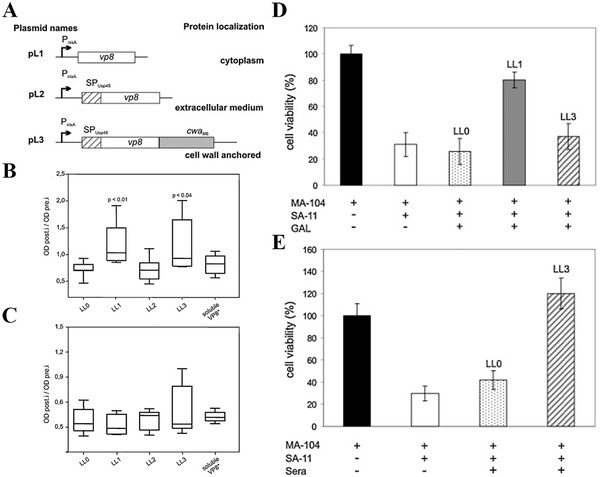
*L. lactis* producing VP8 viral antigen to different cellular locations influence the immunogenicity. (LL1: intracellular form, LL2: secreted form, and LL3: cell wall‐anchored form) (A) Schematic representation of expression element for VP8* protein production. (B) Levels of specific anti‐ VP8* IgA in GAL of day 42. (C) Specific anti‐ VP8* IgG antibody in serum of day 42. (D) GAL or (E) serum from vaccinated mice inhibit SA‐11 virus infection in MA‐104 cells. Adapted with permission.^[^ ^23^ ^]^ Copyright 2011, Elsevier

For mucosal immunization, both intracellular and cell‐wall anchored expression of V8 antigen induces the IgA secretion in gastrointestinal lavage, inhibiting the SA‐11 virus infection in vitro, but only V8 antigen presented in the cell wall could induce serum IgG antibodies after oral immunization (Figure 2).^[^ ^23^ ^]^ The E7 antigen located in the cell surface elicited higher levels of IL‐2 and IFN‐γ cytokines than intracellular and secreted strain following intranasal immunization.^[^ ^24^ ^]^ Another study revealed that the maximum levels of anti‐TTFC antibodies were observed^[^ ^22^ ^]^ when mice were intranasal and intragastric immunized with *L. plantarum* expressing TTFC antigen intracellularly. Thus, the best location of antigens has so far proved inconclusively, and it might be related to the nature of the antigen itself and the routes of immunization.

In summary, different cellular locations of foreign antigens indeed have a critical influence on immune responses. However, the current research regarding the best location of expressed antigens is contradictory. The surface‐localized antigens successfully induced a strong immune response might be due to the fusion of the antigen and the surface display protein, anchoring on the surface of the bacterial cell wall, which exerts an adjuvant effect and enhances the immunogenicity of the antigen.^[^ ^21^ ^]^ On the contrary, the antigen displayed on the surface failed to elicit an immune response might be related to the inappropriate use of signal peptides and anchoring proteins, which leads to insufficient expression and display of the antigen on the cell surface.^[^ ^22^ ^]^ Some antigens expressed in cytoplasm can reach a higher level of synthesis, which might result from the characteristics of the antigen itself and the expression system, thereby causing the most robust immune responses. Besides, a study explained that the reason for the low immunogenicity of the antigen expressed intracellularly was that the antigen was intracellular and isolated from the immune system.^[^ ^20^ ^]^

## 3 THE INFLUENCE OF ADMINISTRATION ROUTES ON IMMUNE RESPONSES

Most of the LAB vaccines are administered to prevent pathogens invaded from the mucosal surfaces such as the gastrointestinal, respiratory, and genitourinary tracts. In order to activate the mucosal and systemic immune system to resist pathogens infection efficiently, LAB vaccines generally are immunized through the mucosa, where presents abundant mucosal‐associated lymphoid tissues. Antigens expressed intracellularly in LAB or secreted from LAB carriers interact with mucosal epithelium, and can be taken up by DCs, M cells, or epithelial cells and transport to the mesenteric lymph nodes, where DCs can directly prime T‐cell responses.^[^ ^12^ ^]^ The naïve B cells can be activated by antigen stimulation and cytokines released by APCs and effector CD4^+^T cells and then differentiate into IgA^+^B cells. The resulting effector IgA^+^B cells traffic to lamina propria, where they differentiate into plasma cells that further secrete high‐affinity IgA.^[^ ^25^ ^]^ SIgA participates in local immunity and has crucial functions in defending pathogens invasion from gastrointestinal, respiratory, and genitourinary tracts. The following review will discuss the different mucosal delivery routes of LAB, including oral, intranasal, and vaginal administration, and the characteristics and immune responses of different immune routes are listed in Table 1

**TABLE 1:** The characters and immune responses of different administration routes

**Administration route**	**Specific characters**	**Vaccine target**	**LAB strain**	**Antigen (location)**	**Immune responses**	**Ref**
Oral	Have to resist drastic pH gradient changes; induce intestinal immune responses and antibody response in other mucosa sites	Helicobacter pylori	*Lactococcus lactis*	NapA (cell‐wall anchored)	NapA‐specific serum IgG and fecal SIgA	^[^ ^29^ ^]^
		Helicobacter pylori	*Lactococcus lactis*	NapA (cytoplasmic)	Anti‐NapA IgG in sera and SIgA in feces	^[^ ^30^ ^]^
		SARS‐CoV	*Lactobacillus casei*	Spike protein SA and SB (cell‐wall anchored)	IgG, IgG1, IgG2a, and IgG2b in sera and IgA antibody in intestinal lavage fluids	^[^ ^38^ ^]^
Intranasal	Lower doses and fewer times compared with oral vaccination; induce SIgA response in lung and vaginal lavage fluids	Streptococcus pneumoniae	*Lactococcus lactis*	PspA (cytoplasmic)	Anitigen‐specific IgG, IgG1, and IgG2a antibodies in serum	^[^ ^32^ ^]^
		SARS‐CoV	*Lactobacillus casei*	Spike protein SA and SB (cell‐wall anchored)	IgG, IgG1, IgG2a, and IgG2b in sera and IgA antibody in lung lavage fluids	^[^ ^38^ ^]^
Vaginal	Induce antibody response in vaginal mucosa	Contraceptive (anti‐ human chorionic gonadotrophin‐β antibody)	*Lactobacillus casei*	hCGβ (cytoplasmic)	Antibody response in serum and vaginal lavage	^[^ ^36^ ^]^

### 3.1 Oral immunization

The intragastric administration is the most common route for LAB vaccines, which can elicit intestinal mucosal immune responses. Most of LAB strains can resist the acid environment of the stomach and high concentration of bile salt, which enables them to reach the intestine alive. Besides, certain *Lactobacillus* strains have the ability to adhere to the surface of mucosal membranes and colonize in the digestive tract as probiotic bacteria.^[^ ^26^ ^]^ All of the properties support LAB as carriers for oral vaccines. *Helicobacter pylori (H. pylori)* colonizing in the gastric tract is the leading cause of gastritis, duodenal ulcer, and even gastric cancer.^[^ ^27^ ^]^ Antibiotic resistance of *H. pylori* makes it urgent to emerge an effective vaccine against *H. pylori*. Recently, numerous studies have investigated the possibility of LAB as an oral vaccine delivery vehicle against *H. pylori* infections. The antigen‐specific serum IgG and fecal SIgA were observed in mice orally given *L. lactis* expressing various antigens of *H. pylori*.^[^ ^28^ ^]^ After *H. pylori* challenges, LAB vaccine provided sufficient protection to immunized mice that decrease the gastric *H. pylori* colonization and urease activity.^[^ ^29^, ^30^ ^]^

### 3.2 Intranasal immunization

Intranasal vaccination can stimulate the immune response in nasal‐associated lymphoid tissue and effectively elicit systemic immunity and mucosal immunity in gastric, respiratory, and reproductive tracts. Compared with oral immunization, nasal immunization avoids gastrointestinal proteolysis and acidic environment challenges, so the dose of antigen and adjuvant required is much lower. Numerous researches have demonstrated that the nasal immunization of the LAB vaccines provided effectively protection against respiratory pathogens like *Streptococcus pneumoniae*.^[^ ^31^ ^]^ Nasally vaccination with a strain of *L. lactis* that expressed pneumococcal surface protein A (PspA) successfully defensed against *Streptococcus pneumoniae* challenge.^[^ ^32^ ^]^ Mice intranasally immunized with recombinant *L. lactis* were striking protected even better than PspA/Alum vaccinated subcutaneously after intraperitoneal and respiratory pneumococcal challenge, although the PspA‐specific IgG antibody elicited by the *L. lactis* vaccine was lower. The significant protection might be attributed to a more balanced IgG1and IgG2a ratio, indicating the immune response shift to Th1‐type immunity that is also critical in eliminating bacterial infections.

### 3.3 Vaginal immunization

Vaginal mucosal tissue also has a large number of immune cells like the intestine and nasal cavity.^[^ ^33^ ^]^ Vaginal administration is another route of mucosal immunization, which has critical functions in preventing transmission of pathogens that infect the body via the vagina such as human immunodeficiency virus (HIV). Researches have revealed that vaginal immunization can induce high titers of antigen‐specific antibodies at mucosal sites and cellular immune responses.^[^ ^34^ ^]^ As early as 1998, Medaglini et al. have reported that 85% of mice were colonized after vaginal administration with one dose of *L. casei* and the colonization even lasted for 10 weeks,^[^ ^35^ ^]^ which provided the possibility for inoculating LAB‐based vaccines through vagina route. After that, a recombinant *L. casei* strain that expressed human chorionic gonadotropin (hCG) was vaginally immunized to mice to induce anti‐hCG antibody responses in vaginal mucosa. Besides, vaginal inoculation with *L. casei* expressing hCG also induced a serum antibody response and splenic lymphocyte proliferation.^[^ ^36^ ^]^ However, the research on vaginal inoculation of LAB vaccine is relatively limited, and further investigations are needed to explore their potentials to generate immune responses.

### 3.4 Comparison of intragastric and intranasal immunization

Both intragastric and intranasal inoculation can elicit mucosal and systemic immunity, but which one is most effective in protecting the host from pathogens invading is still unclear. Several studies have compared the impact of different immune pathways on the immune response. After vaccinated intranasally with lower doses and fewer times than vaccinated orally of *L. lactis* expressing TTFC protein,^[^ ^37^ ^]^ a similar anti‐TTFC serum IgG titers and serum antibody isotype profiles (IgG1, IgG2a, IgG2b, IgG3, IgA, and IgM) were assessed. The TTFC‐specific fecal IgA response was first observed in mice orally inoculated, but a high and steady level of mucosal IgA was detected following intranasal vaccination. Furthermore, intranasal and oral immunization showed equal protection of mice against lethal challenge by injected subcutaneously with tetanus toxin. Similarly, the *L. casei* strain expressing recombinant fusion proteins of spike protein segments SA and SB of SARS‐CoV on the cell surface vaccinated to mice orally or intranasally.^[^ ^38^ ^]^ For the oral route, mice were inoculated via intragastric lavage with recombinant *L. casei* strain. A lighter schedule was employed for intranasal immunizations that mice were administered via nostrils with equal mixtures of live *L. casei* strain. With respect to systemic immune response, there were distinct differences in anti‐SARS S serum IgG antibody and antigen‐specific serum antibody isotypes between the intragastric‐ and intranasal‐ immunization groups. For mucosal immune responses, oral and intranasal immunization elicited high levels of mucosal IgA in intestines and in bronchoalveolar lavage fluids, respectively. However, the potent SARS pseudovirus‐neutralizing activity was only detected in intestinal IgA from mice orally treated. Therefore, it is more likely to induce a mucosal immune response at the locality of inoculation.

## 4 STRATEGIES FOR IMPROVING THE IMMUNOGENICITY OF LAB VACCINES

Although LAB have a certain degree of immunogenicity, sometimes the immune response stimulated by LAB vaccines is not enough to protect the body from pathogens infection. Therefore, researchers have adopted various strategies for LAB vaccines to enhance humoral and cellular immunity and regulate the type of immune responses. The mechanism of these vaccine strategies and immune responses are summarized in Table 2.

**TABLE 2:** The strategies applicated in LAB vaccines to augment the immune responses

**Vaccine strategies**	**Mechanism**	**LAB strain**	**Antigen**	**Administration route**	**Immune responses**	**Protection**
Co‐expression of targeting molecules						
Dendritic cells targeting peptides and antibodies: DCpep, scFv‐CD11c, scFv‐DEC205	Promote the maturation of DCs and enhance antigen uptake by DCs.	*Lactobacillus casei*	E2 protein of bovine viral diarrhea virus	Oral	Serum antibody, mucosal antibody in intestinal mucus and feces, neutralizing activity and cytokines of IFN‐γ and IL‐4	Eliminate the virus from intestine, lung, blood, and spleen.^[^ ^40^ ^]^
		*Lactobacillus plantarum*	NP and M1 protein of avian influenza virus	Oral	Fecal and BALF IgA antibody and IFN‐γ‐producing T‐cells	Provide 80% and 60% protection rate against homologous and heterologous virus infection, respectively.^[^ ^42^ ^]^
		*Lactobacillus plantarum*	Hemagglutinin protein of Influenza A virus (H1N1)	Oral	Serum IgG, BALF and fecal IgA, neutralizing antibody, B220^+^IgA^+^ B cells in PP and IFN‐γ‐producing T‐cells	Increase survival of immunized mice after influenza virus challenge.^[^ ^43^ ^]^
M cells targeting peptides or proteins: Col, CKS9, OmpH.	Enhance the effectiveness of antigen delivery through the intestinal mucosa.	*Lactobacillus saerimneri*	OmpC and FimA proteins of avian pathogenic *Escherichia coli*	Oral	Antigen‐specific serum IgG and SIgA in cecum, nasal cavity, and feces	80% immunized chicken survived after avian E. coli challenges.^[^ ^47^ ^]^
		*Lactococcus lactis*	Viral capsid protein (VP)2 antigen of infectious bursal disease virus	Oral Intramuscular	Neutralizing antibody and mucosal SIgA antibody	Provide complete protection by injection and 80% protection rate by oral.^[^ ^51^ ^]^
Intestinal epithelial cells targeting proteins: FnBPA	Improve the delivery ability and increase antigen uptake by lymphocytes	*Lactococcus lactis*	E7 antigen of human *papillomavirus*16	Intranasal	IgG2a systemic and IgA mucosal immune responses and CTL	Reduce tumor development.^[^ ^56^ ^]^
Cytokines						
IL‐12	Stimulate activity of cytotoxic T lymphocytes and natural killer (NK)‐cells; promote IFN‐γ production.^[^ ^57^ ^]^	*Lactococcus lactis*	E7 antigen of human *papillomavirus*16	Intranasal	ELISPOT: IFN‐γ‐secreting T‐cells^[^^]^; E7‐specific CTL response, cytokines of IL‐12 and IFN‐γ^[^ ^59^ ^]^	Increase survival rates and regress tumor growth.^[^ ^58^, ^59^ ^]^
		*Lactococcus lactis*	Leishmania antigen LACK	Oral	IgA antibodies in intestinal washes and a systemic Th1 immune response	No report^[^ ^60^ ^]^
IL‐2	Induce the proliferation of immune cells and the differentiation of effector CD4^+^T cells.^[^ ^63^ ^]^	*Lactococcus lactis*	UreB protein of *Helicobacter pylori*	Oral	Serum antibody, fecal IgA and serum IFN‐γ, IL‐4, and IL‐17	Accelerate H. pylori clearance and reduce the levels of H. pylori colonization in the stomach.^[^ ^65^ ^]^
IL‐6	Promote the secretion of antibodies by maturated B cells and the production of IL‐2.^[^ ^68^ ^]^	*Lactococcus lactis*	Tetanus toxin fragment C	Intranasal	anti‐TTFC IgG and IgA responses	No report^[^ ^69^ ^]^
		*Lactococcus lactis*	*Brachyspira* membrane protein B	Oral	Serum IgG, IgG1, and IgG2a, feces and intestinal IgA, IL‐4, IFN‐γ, and IL‐2‐secreting lymphocytes in PPs	No report^[^ ^49^ ^]^
Mucosal adjuvant						
Cholera toxin	Induce the polarization of T cells towards Th2 and B cells activated and isotype switching.	*Lactococcus lactis*	Influenza virus nucleoprotein	Oral	Serum antibody, mucosal IgA in intestine and upper respiratory washes, and IFN‐γ and IL‐4 secreting splenocytes	Improve the resistance to divergent influenza viruses challenge.^[^ ^74^ ^]^
		*Lactobacillus casei*	Matrix protein‐2 of influenza virus	Oral Intranasal	Serum IgG, IgA antibody in lung and intestine, and IFN‐γ and IL‐4 secreting splenocytes	Decrease virus titers in the lung tissues after challenge with influenza virus.^[^ ^76^ ^]^
Heat‐labile enterotoxin	Induces mixed Th1 and Th2 immune responses and B cells and DCs activation.	*Lactobacillus casei*	VP4 antigen of porcine rotavirus	Oral	Serum IgG, specific IgA in ophthalmic, vaginal wash and feces, neutralization activity	No report^[^ ^80^ ^]^
		*Lactobacillus casei*	F4 (K88) fimbrial adhesin FaeG of enterotoxigenic *Escherichia coli*	Oral	SIgA in feces, vaginal lavage, and nasal lavage, serum antibody, and lymphocyte proliferation	Provide complete protection to immunized mice.^[^ ^83^ ^]^
Flagellin	Toll‐like receptor 5 agonist, stimulate the activation of NF‐kB, the maturation of DCs^[^ ^85^ ^]^	*Lactobacillus acidophilus*	Gag protein of HIV‐1	Oral	IgA‐secreting and IFN‐γ‐producing lymphocytes from Peyer's patches, female reproductive tract, and large intestine	No report^[^ ^87^ ^]^
Polymer's coating						
Enteric‐coated capsules	Improve the stability and viability of LAB in the gastrointestinal tract	*Lactococcus lactis*	Hemagglutinin protein of H5N1	Oral	Serum IgG and fecal IgA, IFN‐γ secreting splenocytes	Provide full immune protection against H5N1 virus challenges^[^ ^96^ ^]^
Alginate/chitosan/alginate microcapsules		*Lactobacillus plantarum*	*Brachyspira* membrane protein B	Oral	Serum IgG, IgG1, and IgG2a, feces, and intestinal IgA	No report^[^ ^97^ ^]^

### 4.1 Co‐expression of targeting molecules

#### 4.1.1 Dendritic cells targeting peptides or antibodies

Dendritic cells (DCs) are professional antigen‐presenting cells (APCs) and are exclusive APCs that can active naïve T cells. They play a critical role in initiating, regulating, and maintaining immune responses in the host. In the mucosal system, DCs are widely distributed in or beneath the epithelium. They can phagocytose antigens through passing protrusions between epithelial cells or sample antigens that cross the epithelial layer via M cells and then traffic to lymphoid tissues, where possessed antigens are presented to T cells to directly prime T‐cell responses and B‐cell differentiation into plasma cells. These properties suggest targeting DCs to improve antigen delivery efficiency is an attractive strategy for triggering effective mucosal immune responses.

To date, numerous studies have demonstrated that expression antigens fused with DCs targeting peptides or antibodies can augment immune responses. There are many special markers on the surface of DCs such as CD11c and DEC205. LAB has been engineered to express a 12‐mer DC‐binding peptide (DCpep), single‐chain variable fragment against CD11c(scFv‐CD11c) and DEC 205(scFv‐DEC205), which could enhance the internalization of DCs effectively.

**FIGURE 3:**
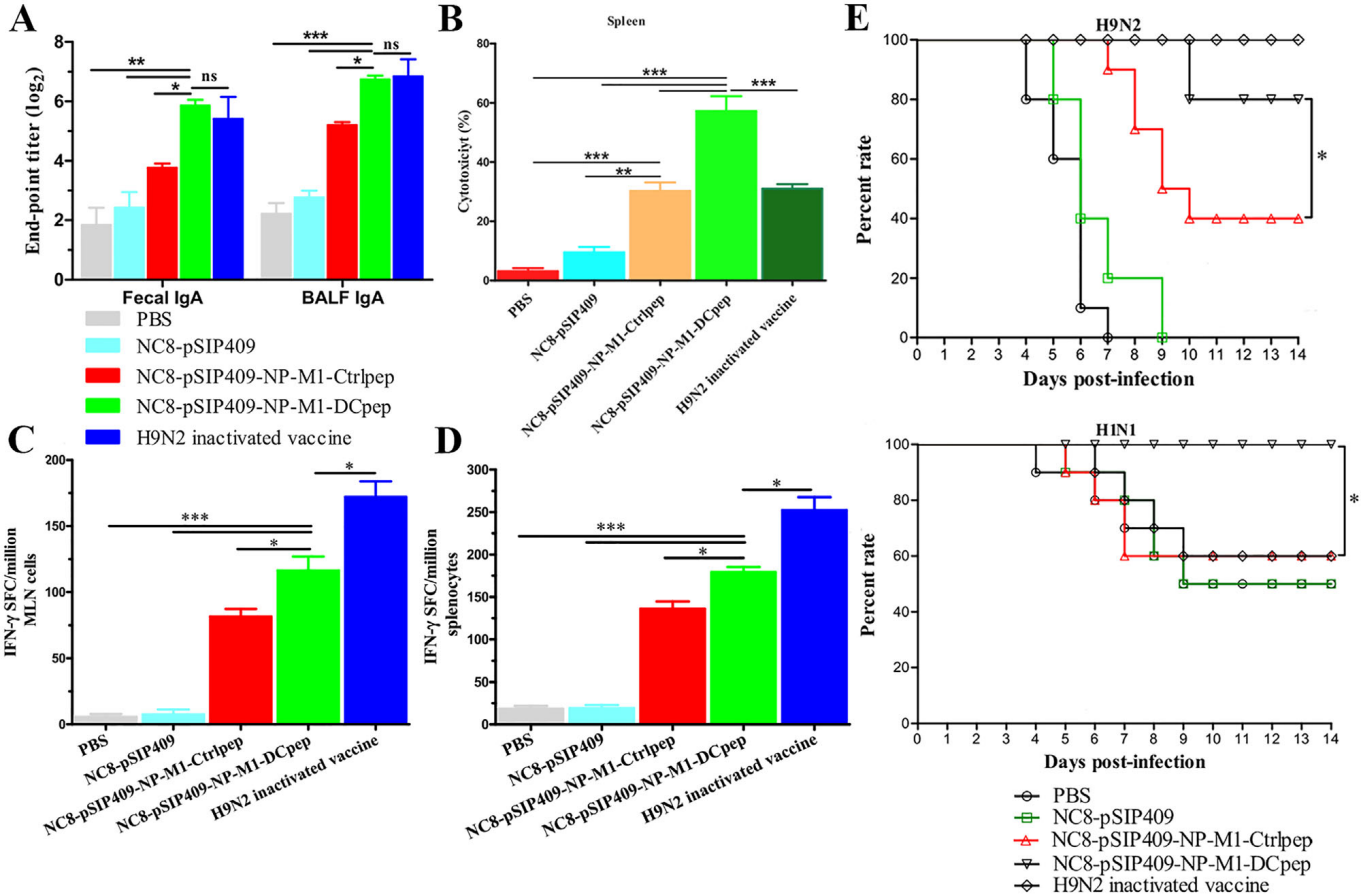
*L. plantarum* co‐expressing NP and M1 antigens of avian influenza virus and DCpep protect mice from homologous and heterologous influenza virus infections. (A) The antigen‐specific sIgA titers in feces and BALF of day 44 after the primer vaccination. (B) NP and M1‐specific CTL response. Numbers of IFN‐γ‐secreting cells in (C) MLN cells and (D) splenocytes. (E) The survival rate of H9N2 and HIN1 infected after vaccination. (*P < 0.05, **P < 0.01, and ***P < 0.001). Adapted from Creative Commons CC BY open access publications.^[^ ^42^ ^]^Copyright 2016, The Author(s)

A 12‐mer DC‐targeting peptide (FYPSYHSTPQRP),^[^ ^39^ ^]^ derived from a phage display library, can upregulate the expression levels of mature molecules CD40, CD80, and CD86 on the surface of DCs.^[^ ^40^ ^]^ Huang et al. have indicated that *L. plantarum* expressing the fusion protein of S‐DCpep induced high titer of antigen‐specific serum and mucosa antibodies, as well as serum and feces‐neutralizing antibodies against PEDV virus.^[^ ^41^ ^]^ Besides, mice orally immunized with influenza virus antigen and DCpep elicited not only elevated antigen‐specific antibodies but relatively high levels of Th1 and CTL responses was induced, which protected 80% immunized mice from homologous influenza virus infection and 60% mice from heterologous influenza virus infection (Figure 3).^[^ ^42^ ^]^ The single‐chain variable fragment against CD11c (scFv‐CD11c) is a useful DCs targeting antibody. *L. plantarum* carrying HIN1 antigen and an anti‐CD11c antibody promoted the maturation of DCs, the proliferation of T cells, and the production of robust neutralizing antibodies, providing adequate protection against influenza viruses challenge.^[^ ^43^ ^]^ A few works have reported that targeting DCs with anti‐DEC 205 single‐chain antibody in LAB. It revealed that *L. plantarum* expressing a recombinant ScFv against DEC 205 on their surface increases their internalization by DCs.^[^ ^44^ ^]^ There is no related work showing the influence of LAB expressing antigen fused with ScFv‐DEC205 to immune effect.

#### 4.1.2 M cells targeting peptides and proteins

Microfold cells located in follicle‐associated epithelium have distinguished morphological features from other epithelial cells. There is no a mucus layer on their apical surface, but replaced by micro folds composed of short and irregular microvilli.^[^ ^45^ ^]^ The basolateral side of M cells has a deep membrane invagination, and different types of lymphocytes are enveloped inside, including T cells, B cells, DCs, and macrophages. These characteristics of M cells enable them to efficiently phagocytize pathogenic microorganism antigens from the intestinal lumen and then transport them to mucosal lymphoid tissues via DCs or macrophages in the basolateral pocket. In addition, M cells are essential in priming mucosal immune responses, especially the production of secretory IgA antibodies, which have a vital function in defending against pathogenic microorganisms through mucus. For oral vaccine, delivering antigen directly to M cells represents an ideal strategy to achieve systemic and mucosal immune responses.

*L. casei* expressing Col, an M cell‐targeting peptide with the neutralizing epitope region of porcine epidemic diarrhea virus (PEDV) was identified to induce antigen‐specific sIgA antibodies in external genital tract, intestinal mucus, and feces, suggesting a more effective sIgA‐based mucosal immunity.^[^ ^46^ ^]^ Similarly, chickens orally administered with recombinant *Lactobacillus* strains activated high serum IgG antibody and mucus IgA antibody responses, providing 80% protection rate against pathogenic *Escherichia coli* infections.^[^ ^47^ ^]^ Besides Col peptide, CKS9 is another M cell‐targeting peptide derived from a phage display screening, which consists of 9‐amino acids. Previous work has indicated that the conjugation of CKS9 to chitosan nanoparticles could enhance binding affinity to M cells and target follicle‐associated epithelium in vivo.^[^ ^48^ ^]^ Subsequently, *L. lactis* as a vector expressing murine IL‐6 conjugated with CKS9 was reported, which induced Th2‐based IgG1, Th1‐based IgG2a, and feces IgA antibody responses.^[^ ^49^ ^]^ Unlike the targeting peptide, the outer membrane protein H (OmpH) of *Yersinia enterocolitica* is the ligand for complement C5a receptor which shows highly expressed levels on the apical surface of M cells.^[^ ^50^ ^]^ The capsid protein of infectious bursal disease virus with OmpH in *L. lactis* had proven to increase neutralizing and sIgA antibody titers, resulting in complete protection by injection immunization and 80% protection rate by oral immunization.^[^ ^51^ ^]^ The same LAB strain co‐expressing enterotoxin protein and OmpH also provided comprehensive protection against enterotoxigenic *Escherichia coli* infections.^[^ ^52^ ^]^

#### 4.1.3 Intestinal epithelial cells targeting proteins

The intestinal epithelium is composed of a single layer of cells, which plays an essential role in resisting the invasion of pathogens in innate immunity. Meanwhile, intestinal epithelial cells can transport antigens from the intestinal lumen across epithelium and present to immune cells.^[^ ^53^ ^]^ Therefore, antigens targeting intestinal epithelial cells may increase their uptake by lymphocytes, thereby inducing an effective immune response.

The primary way to target intestinal epithelial cells is to display fibronectin‐binding protein A (FnBPA) on the surface of LAB strains. FnBPA has been identified on the cell surface of Staphylococcus aureus, which mediates the adhesion and invasion of bacteria to epithelial cells via the interaction of FnBPA and α5β1 integrins presented in the host cell membrane.^[^ ^54^ ^]^ One research had shown that the presence of FnBPA could trigger the activation of bone marrow DCs and the differentiation of DCs in mesenteric lymph node and Peyer's patch,^[^ ^55^ ^]^ which may be attributed to promoting the uptake of antigen by DCs. In addition, mice intranasally administered with E7 antigen from human *papillomavirus*16 (HPV16) and FnBPA have induced humoral Th1 systemic immune response and IgA mucosal immune response. After challenge with TC‐1 tumor cells, FnBPA with HPV antigen could significantly decelerate the growth rate of tumors.^[^ ^56^ ^]^

### 4.2 Co‐expression of cytokines

Cytokines, synthesized and secreted by immune cells and non‐immune cells, have a wide range of biological activities. They participate in immune response and immune regulation and trigger innate immunity as well as adaptive immune response. Due to the crucial function of cytokines in resisting pathogenic microbial infections and inhibiting tumor growth, several cytokines, including IL‐12, IL‐2, and IL‐6, have been investigating as LAB vaccine booster to elicit systemic and mucosal immune responses.

The pro‐inflammatory cytokines IL‐12 are mainly produced and secreted by DCs, transformed B cells, and macrophages. IL‐12 function in stimulating the activity of cytotoxic T lymphocytes and natural killer (NK)‐cells, and promoting IFN‐γ production, which further facilitates additional APCs production of IL‐12 and Th1 differentiation.^[^ ^57^ ^]^ Many reports have characterized the effect of IL‐12 as an immunoregulatory cytokine during infection and cancer. The LAB vaccines expressing E7 antigen from HPV16 had been intranasally immunized mice with another LAB strain secreting IL‐12 cytokine or co‐expressing antigen and IL‐12.^[^ ^58^, ^59^ ^]^ The E7 antigen with IL‐12 effectively induced higher levels of IFN‐γand more robust antigen‐specific CTL responses in splenocytes. Compared to the *L. lactis* only expressing E7 antigen vaccinated group, the tumor volume of mice treated with E7 antigen and IL‐12 was significantly smaller, and *L. lactis* co‐expressing antigen and IL‐12 could further regress tumor growth.^[^ ^59^ ^]^ Additionally, IL‐12 was expressed by *L. lactis* to defense *Leishmania major* infection with LACK antigen.^[^ ^60^, ^61^ ^]^ Oral vaccination with LACK and IL‐12 was capable of eliciting the secretion of IgA in intestinal washes and IFN‐γ in splenocytes and mesenteric lymph node cells, indicated eliciting mucosal and systemic Th1 immune response.

IL‐2 is a significant regulatory cytokine primarily produced by CD4^+^ helper T (Th) cells when activated by antigen^[^ ^62^ ^]^ and produced by CD8+ T cells, NK cells, natural killer T cells, activated DCs, and mast cells but at low levels. IL‐2 acts as growth factor that can induce the proliferation of various immune cells, including T cells, NK cells, and B cells. It also modulates effector CD4^+^ T cells for Th1 cell differentiation and Th2 cell differentiation^[^ ^63^ ^]^ while conversely inhibiting the development of Th17 cells. In addition, IL‐2 can increase the cytolytic activity of NK cells and lymphokine‐activated killer cells.^[^ ^64^ ^]^ *L. lactis* was engineered to express IL‐2 and UreB antigen for preventing *H. pylori* infection.^[^ ^65^ ^]^ The addition of IL‐2 increased anti‐ *H. pylori* serum IgG and feces sIgA levels and promoted the secretion of relative immune factors such IFN‐γ and IL‐4. More importantly, IL‐2 as an adjuvant for the LAB vaccine could accelerate *H. pylori* clearance and reduce the levels of *H. pylori* colonization in the stomach after challenging with *H. pylori*.

IL‐6, produced by many cell types including immune cells and stroma cells,^[^ ^66^ ^]^ is involved in almost every aspect of the innate and adaptive immune system with broad‐ranging effects.^[^ ^67^ ^]^ IL‐6 effectively influences the synthesis and secretion of acute‐phase proteins that have fundamental roles in integrated host defenses against viruses, parasites, and bacterial infections. Besides, IL‐6 can promote maturated B cells to secrete antibodies and regulate IL‐2 receptors to promote IL‐2 production.^[^ ^68^ ^]^ One study showed mice vaccinated in the presence of IL‐6 elicited higher anti‐TTFC IgG and IgA titers.^[^ ^69^ ^]^ In another separated study, *Brachyspira* membrane protein B (M‐BmpB) with *L. lactis* IL1403 expressing IL‐6^[^ ^49^ ^]^ also increased serum IgG and intestinal IgA secretion and the amount of lymphocytes secreting IFN‐γ and IL‐2, which was associated with the proliferation and differentiation of T cells.

### 4.3 Combination with bacterial toxins and flagellin

Most LAB vaccines are immunized via the mucosal routes. LAB alone only expressing antigens with low immunogenicity cannot trigger an effective immune response. Therefore, adjuvants are needed to be co‐delivered with the LAB vaccines to elicit strong innate and adaptive immune responses for providing efficient protection against pathogens infection. For mucosal vaccines, commonly used adjuvant may be disable in improving immunogenicity, such as aluminum (alum) adjuvant, which has been widely applied in issued vaccines. On the contrary, the TLR agonist, cholera toxin (CT), and heat‐labile enterotoxin (LT) are investigated as potential adjuvants for mucosal vaccines.

The adjuvant cholera toxin derived from Vibrio cholera has an AB_5_ structure composed of an enzymatically ADP‐ribosylating active A subunit non‐covalently linked with a pentameric oligomer receptor‐binding B subunits.^[^ ^70^ ^]^ After pentamer B subunits binding with GM1‐ganglioside on the surface of mammalian cells, the A subunit enters the target cell cytosol and transfers the ADP‐ribose group to a GTP binding‐protein, causing the activation of adenylate cyclase and the intracellular accumulation of Camp.^[^ ^71^ ^]^ The CT administered with antigens induces the polarization of T cells towards Th2 and B cells activation and isotype switching. It also stimulates the production of cytokine profiles related to Th2‐type immune responses.

CTB enhances mucosal immune responses by promoting antigens across mucosal surfaces and improving the affinity between antigens and immune cells. A series of studies have demonstrated that the CTB could be an adjuvant via the oral or nasal administration routes in LAB vaccines against influenza viruses, *Bordetella pertussis*, and *H. pylori* infections.^[^ ^72^ ^]^ Influenza virus proteins (hemagglutinin and nucleoprotein) with CTB adjuvanted^[^ ^73^, ^74^ ^]^ were capable of producing antibodies to influenza virus proteins and IFN‐γ and IL‐4‐secreting lymphocytes, which inhibited the activity of influenza viruses. Besides, the *L. lactis* expressing nucleoprotein with CTB could protect the immunized mice from a lethal dose of divergent influenza viruses (H1N1, H3N2, and H5N1) infected. Another study described a fusion protein of multiple epitopes of UreA and UreB with CTB expressed in *L. lactis*.^[^ ^75^ ^]^ Intragastric treatment with the fused protein leaded B cells to secret antibodies, which could reduce the activity of urease and the number of bacteria in the stomach. The active subunit A of CT (CTA1) conjugated with influenza virus antigens was displayed on the surface of *L. casei* against divergent influenza subtypes infection.^[^ ^76^, ^77^ ^]^ The mucosal adjuvant of CTA1 enhanced the humoral and cellular immune responses, as well as mucosal immune responses, providing higher survival rate and lower virus titers in the lung and even long‐lasting protection after challenges with divergent influenza viruses, including H5N2, H1N1, H5N1, and H9N2.

The heat‐labile enterotoxin expressed by enterotoxigenic *Escherichia coli* (ETEC) strains has 80% amino acid homology and a similar structure with CT.^[^ ^78^ ^]^ But there are distinct differences between CT and LT in the immunologic biases that LT induces mixed Th1 and Th2 immune responses, Th17 cells differentiation, B cells, and DCs activation, and cytokine secretion.^[^ ^79^ ^]^ Some works have reported that mice orally immunized with *L. casei* expressing LTB conjugated with antigens from porcine rotavirus and porcine epidemic diarrhea virus (PEDV) respectively^[^ ^80^, ^81^ ^]^ showed higher systemic and mucosal antibodies and neutralization ability than those immunized without LTB. Similarly, after intragastric immunization with *L. plantarum* that surface displayed HA2‐LTB against H9N2 influenza virus,^[^ ^82^ ^]^ LTB significantly increased the percentages of CD4^+^ T cells secreting IL‐4, IFN‐γ, and IL‐17 and CD8^+^ T cells secreting IFN‐γ in spleen and mesenteric lymph nodes, which was associated with the protection against H9N2 influenza virus. In addition to fused expression with antigens, LT can be applied as an adjuvant in combination with LAB vaccines. One research investigated the immunogenicity of the antigen of enterotoxigenic *Escherichia coli* (ETEC) combined with co‐expressed non‐toxic LTA (LTAK63) and LTB or fused‐expressed LTAK63 and LTB by *L. casei*. The high expression of serum IgG and mucosal IgA, as well as the complete protection, were observed in mice orally vaccinated with co‐expressed LTAK63 and LTB.^[^ ^83^ ^]^

Toll‐like receptor 5 (TLR5) is widely distributed in many types of immune cells and epithelial cells^[^ ^84^ ^]^ and is critical in triggering innate immunity and priming acquired immunity. Flagellin is the structural component of bacterial flagella that acts as a TLR5 agonist mainly through the MyD88‐dependent signaling pathway.^[^ ^85^ ^]^ The flagellin fused or co‐administered with antigens has shown tremendous potency as an adjuvant in stimulating the activation of NF‐kB, the maturation of DCs, and the induction of NF‐kB‐mediated cytokines and chemokines, which has been proved by a recombinant *L. gasseri* strain expressing flagellin. Previous work has indicated that recombinant‐flagellin‐OVA fusion protein could evoke robust antigen‐specific immune responses without the help of additional adjuvants while OVA individually cannot.^[^ ^86^ ^]^ Additionally, *Lactobacillus acidophilus* as a vector displaying HIV‐1 Gag and flagellin has been developed.^[^ ^87^ ^]^ It significantly elevated NF‐kB activation, myeloid DCs maturation markers, and the count of lymphocytes secreting Gag‐specific IgA and lymphocytes producing IFN‐γ following immunization with LAB displaying Gag and flagellin.

### 4.4 Polymer coating improves the efficiency of LAB oral vaccines

Oral delivery of vaccines holds the characteristics of simplicity, non‐invasiveness, and high compliance and can evoke effective intestinal immune responses to resist pathogens that invade from the intestinal tract. Vaccines delivered by oral routes should pass through the harsh gastrointestinal environment to reach the intestines. Antigens in the gastrointestinal tract have to tolerate and avoid disturbing drastic pH gradient changes and digestive enzymes. Up to now, various encapsulation methods that improve the stability and viability of LAB in hash acid conditions have been developed.^[^ ^88^ ^]^ The most common method protecting LAB is calcium‐alginate coating. The *L. rhamnosus* and *L. acidophilus* encapsulated in alginate microbeads show increased survivability^[^ ^89^ ^]^ and that is further improved when the alginate microbeads are coated by chitosan. Another study reports that *L. casei*‐encapsulated Ca‐alginate as a core and outer Ca‐alginate/protamine composite as a shell^[^ ^90^ ^]^ ensures the bacteria survive in the stomach and rapidly release in the intestine. The chitosan‐alginate is another method to protect LAB under highly acid conditions.^[^ ^91^ ^]^ The layer‐by‐layer assembling of chitosan‐alginate is formed by hydrogen‐bonding and electrostatic adsorption between chitosan and alginate. However, the results show a decreased survival rate of bacteria when the number of layers above 3, which may be associated with the particle size and reduced cross‐linking density of microparticles. In addition, the cellulose acetate phthalate as a wall^[^ ^92^ ^]^ also is used to microencapsulate the L. acidophilus to protect their activity in a harsh environment. Recently, some new coatings that protect *Escherichia coli Nissle 1917* (EcN) from environmental attack in the gastrointestinal tract have been reported, including liposomes,^[^ ^93^ ^]^ enteric materials,^[^ ^94^ ^]^ and polydopamine.^[^ ^95^ ^]^ These coating methods might be adopted for encapsulating LAB in future research.

**FIGURE 4:**
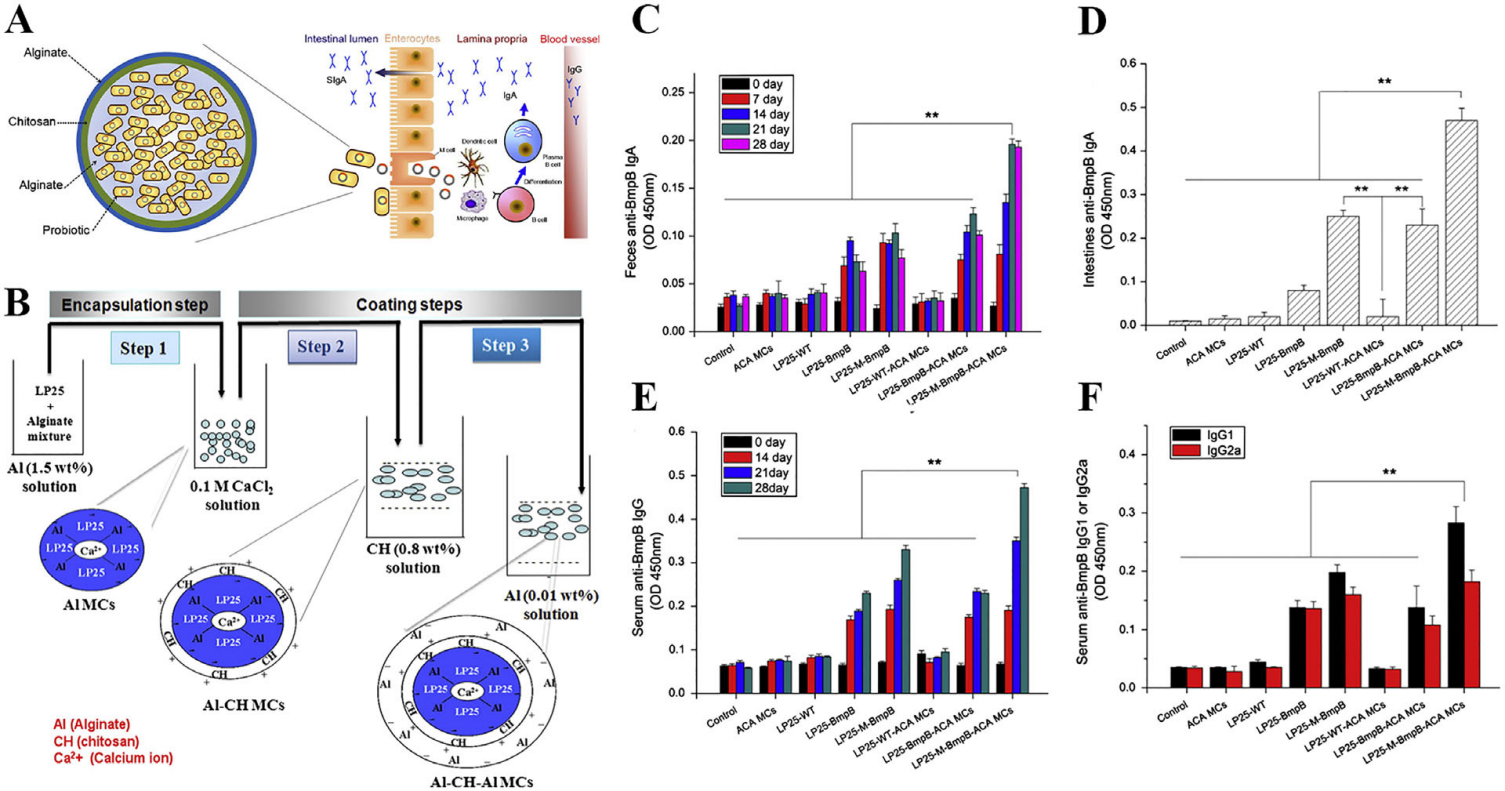
*Lactobacillus* plantarum 25 (LP25) encapsulated alginate/chitosan/alginate microcapsules for effective oral vaccine delivery. (A) Schematic illustration to show the process of inducing mucosal immune response. (B) Preparation of LP25 coated with alginate/chitosan/alginate microcapsules. BmpB‐specific IgA levels in (C) feces and (D) intestines. (E) Serum BmpB‐specific IgG levels. (F) Serum BmpB‐specific IgG1 and IgG1 antibody. (means ± SD, *n* = 5 mice, **p < 0.01). Adapted with permission.^[^ ^97^ ^]^Copyright 2014, Elsevier

Several studies have focused on promoting the immune effect of LAB vaccines by improving the viability of LAB in the gastrointestinal tract. Oral delivery of enteric‐coated recombinant *L. lactis* mini‐capsules^[^ ^96^ ^]^ effectively induced serum and fecal antibodies and cell‐mediated immune responses, providing full protection against lethal challenges of the H5N1 virus. Furthermore, *L. plantarum* expressing BmpB antigen loaded into alginate/chitosan/alginate microcapsules^[^ ^97^ ^]^ significantly prolonged survival during exposure to SGF and SIF in vitro. Thus, the intestinal and systemic immune responses were significantly enhanced after oral administration with the alginate/chitosan/alginate immobilize LAB vaccine (Figure 4).

To summarize, the various strategies discussed here can enhance the immune responses of LAB vaccines, although their mechanisms to improve immunogenicity are different. LAB that expresses targeting molecules to target DCs, M cells, and intestinal epithelial cells can increase the efficiency of antigens delivery through the mucosa and the antigen uptake by APCs, further promoting the maturation of these cells. Similarly, polymer‐encapsulated LAB improves the survival of LAB in the gastrointestinal tract, thereby increasing the uptake of antigen by APCs. Cytokines including IL‐2, IL‐12, and IL‐6 and adjuvants including CT and LT that are expressed by LAB or combined with LAB vaccines mainly induce the proliferation, differentiation, and activation of immune cells and promote the secretion of cytokines, thereby enhancing the antigen‐specific immune response. In addition, the combination of these potential strategies may further enhance the effectiveness of the LAB vaccines, which is worthy to investigate in future research.

## 5 SUMMARY AND PERSPECTIVES

Over the past two decades, LAB have been engineered to be an attractive antigen producer to immunize against a variety of pathogens ranging from bacteria to viruses and parasites. For different antigens, it is necessary to choose a suitable LAB strain as an expression host. The plasmid expression system and expression efficiency of antigens vary with LAB strains, making it difficult to compare the immune effects in these studies. Limited by genetic technology, published studies usually employ *Lactococcus* and *Lactobacillus* as carriers to produce foreign antigens. However, exploring the possibility of other LAB genera like Bifidobacterium, which possesses the characteristics of probiotics and naturally inhabits the intestinal tract, may improve the effectiveness of vaccines delivered through oral routes. Besides, the techniques for expressing foreign proteins in Bifidobacterium are being developed.^[^ ^98^ ^]^ The immunity of the antigen localization in the vector is influenced by the systemic and mucosal administration pathways, but there is no consistent conclusion on which is the best expression position. It may be related to the efficiency of the signal peptide and anchor protein. Both intragastric and intranasal administration can provoke mucosal and systemic immune responses, but intranasal immunization usually requires a lower dose and frequency of antigen administration compared to intragastric immunization.^[^ ^37^ ^]^ Although LAB vaccines have intrinsic adjuvant properties,^[^ ^99^ ^]^ appropriate strategies are usually required to strengthen their immunogenicity.^[^ ^100^ ^]^ For example, increase the uptake of antigen by APCs and promote the maturation of DCs by targeting DCs, M cells, and intestinal epithelial cells or wrapping LAB carrier cells with biological materials. In addition, facilitate the secretion of antigen‐specific antibodies and proliferation of T cells and B cells, including co‐expression or co‐administration of cytokines and adjuvants such as CT and LT. A large number of researches have proved these potential strategies to enhance the immunogenicity of LAB vaccines.

With the continuous deepening of preclinical research on LAB vaccines, several LAB‐based HPV vaccines developed have entered the clinical trials. The early first Phase I/IIa clinical trial study was designed involving patients with HPV16‐positive cervical intraepithelial neoplasia (CIN)3 or 2 orally treated with recombinant *L. casei* expressing mutated HPV16 E7 (GLBL101c) that was enclosed in an enteric capsule. No profound adverse effect was observed in patients on 1, 2, 4, or 6 capsules/day. Oral vaccination of GLBL101c successfully elicited E7‐specific cell‐mediated immune responses in cervical lymphocytes but showed no effect on PBMCs in patients with pathological CIN3 on 4 capsules/day, and 80% of patients experienced pathological downgrade from CIN3 to CIN2.^[^ ^101^ ^]^ However, when GLBL101c was administered to the patients with HPV16‐positive CIN2, the E7‐specific Th1 immune responses and the regression rate from CIN3 to CIN2 or less showed no significant statistical difference between GLBL101c and placebo groups.^[^ ^102^ ^]^ The unsatisfactory clinical trial results were due to limited expression levels of E7 in CIN2 lesion, and weak antigen‐presentation ability of GLBL101c. Therefore, the next generation of *Lactobacillus* expressing HPV16 E7 on the cell surface, which is more immunogenic than GLBL101c, is already under clinical research.^[^ ^103^ ^]^ In addition, there are two other clinical trials of LAB‐based vaccines: one is to explore the safety and humoral immunity of *L. casei* displaying HPV16 E7 protein on the surface in patients with CIN3,^[^ ^104^ ^]^ the other is oral administration of *L. lactis* NZ9000 expressing HPV16 E6 and E7 antigen.^[^ ^105^ ^]^ Although some progress has been made in the development of LAB vaccines, the long‐lasting antibody responses and long‐lived memory T and B cells should be further investigated to acquire sufficient protective immunity.^[^ ^106^ ^]^ Such efforts need to be unremittingly explored to developed LAB‐based delivery of therapeutic and prophylactic molecules.

## CONFLICT OF INTEREST

Xun Sun is a member of the *Exploration* editorial board. The authors declare no conflict of interest.

## AUTHOR CONTRIBUTIONS

Nan Qiao collected the literature materials and wrote the manuscript. Xun Sun, Guangsheng Du, and Xiaofang Zhong revised the manuscript. All of the authors have read and approved the final manuscript.
